# Association of D-dimer level with thrombotic events, bleeding, and mortality in Japanese patients with solid tumors: a Cancer-VTE Registry subanalysis

**DOI:** 10.1007/s10147-024-02475-6

**Published:** 2024-03-02

**Authors:** Mashio Nakamura, Masato Sakon, Mitsuru Sasako, Takuji Okusaka, Hirofumi Mukai, Keiichi Fujiwara, Hideo Kunitoh, Mari S. Oba, Hideo Wada, Jun Hosokawa, Atsushi Takita, Masataka Ikeda

**Affiliations:** 1 Nakamura Medical Clinic, 7-1510, Hidamarinooka, Kuwana, Mie 511-0867 Japan; 2 Department of Gastroenterological Surgery, Osaka International Cancer Institute, Chuo-Ku, Osaka, Japan; 3 Department of Surgery, Yodogawa Christian Hospital, Higashi Yodogawa-Ku, Osaka, Japan; 4 Department of Hepatobiliary and Pancreatic Oncology, National Cancer Center Hospital, Chuo-Ku, Tokyo, Japan; 5 Division of Medical Oncology, National Cancer Center Hospital East, Kashiwa, Chiba Japan; 6 Department of Gynecologic Oncology, Saitama Medical University International Medical Center, Hidaka, Saitama Japan; 7 Department of Medical Oncology, Japanese Red Cross Medical Center, Shibuya-Ku, Tokyo, Japan; 8 Department of Medical Statistics, Toho University, Ota-Ku, Tokyo, Japan; 9 Department of Clinical Data Science, Clinical Research & Education Promotion Division, National Center of Neurology and Psychiatry, Kodaira, Tokyo Japan; 10 Department of General and Laboratory Medicine, Mie Prefectural General Medical Center, Yokkaichi, Mie Japan; 11 Primary Medical Science Department, Daiichi Sankyo Co., Ltd, Chuo-Ku, Tokyo, Japan; 12 Data Intelligence Department, Daiichi Sankyo Co., Ltd, Shinagawa-Ku, Tokyo, Japan; 13 Division of Lower Gastrointestinal Surgery, Department of Gastroenterological Surgery, Hyogo Medical University, Nishinomiya, Hyogo Japan

**Keywords:** D-dimer, Japan, Mortality, Tumors, Venous thromboembolism

## Abstract

**Background:**

The D-dimer test is a simple test frequently used in routine clinical screening for venous thromboembolism (VTE). The Cancer-VTE Registry was a large-scale, multicenter, prospective, observational study in Japanese patients with cancer. This study aimed to clarify the relationship between D-dimer level at cancer diagnosis (baseline) and the incidence of events during cancer treatment (1-year follow-up period).

**Methods:**

This was a post hoc sub-analysis of patients from the Cancer-VTE Registry whose D-dimer levels were measured at baseline. The incidence of events during the 1-year follow-up period was evaluated stratified by baseline D-dimer level. Adjusted hazard ratios for D-dimer level and events during the follow-up period were evaluated.

**Results:**

Among the total enrolled patients, baseline D-dimer level was measured in 9020 patients. The mean ± standard deviation baseline D-dimer level was 1.57 ± 3.94 µg/mL. During the follow-up period, the incidence of VTE, cerebral infarction/transient ischemic attack (TIA)/systemic embolic events (SEE), bleeding, and all-cause death increased with increasing baseline D-dimer level. The incidence of all-cause death increased with increasing D-dimer level regardless of cancer stage. The adjusted hazard ratio of all-cause death was 1.03 (95% confidence interval: 1.02–1.03) per 1.0-µg/mL increase in baseline D-dimer level.

**Conclusions:**

Increases in D-dimer levels were associated with a higher risk of thrombotic events, such as VTE and cerebral infarction/TIA/SEE, during cancer treatment. Furthermore, higher D-dimer levels at cancer diagnosis were associated with a higher mortality rate, regardless of cancer stage.

**Supplementary Information:**

The online version contains supplementary material available at 10.1007/s10147-024-02475-6.

## Introduction

Cancer is a diverse disease, and biomarker assessment at the time of cancer diagnosis is important for identifying the therapeutic target, treatment decisions, prognosis prediction, and risk assessment of complications. D-dimer, a coagulation biomarker, represents fibrin degradation products, and its levels are also known to be elevated in various diseases that have a tendency to form blood clots [1, 2]. In cancer patients, D-dimer values may reflect a different pathology than in non-cancer patients. Cancer patients are known to have elevated D-dimer levels even in the absence of clinically meaningful thrombus, and the mechanism by which this occurs involves the activation of the coagulation cascade by tumor cells [3]. Thus, D-dimer is a highly sensitive but nonspecific biomarker for thrombus, including venous thromboembolism (VTE), in cancer patients.

Several studies have reported an association between high D-dimer levels and poor prognosis in cancer patients [4–13], suggesting that plasma D-dimer levels may have potential as markers of cancer progression. It is of clinical interest that D-dimer, as a biomarker that is commonly measured across cancer types, may help predict prognosis. However, systematic reviews and meta-analyses on this subject have been dominated by small-scale studies conducted retrospectively in a single center [14]. Further, evidence from large-scale, prospective studies, especially in Japan, is insufficient.

The Cancer-VTE Registry was a large prospective, observational study conducted under routine clinical practice in Japan in patients with six types of solid cancer [15–17]. At the time of enrollment in this registry, VTE screening using a combination of D-dimer level measurement and imaging tests was performed to assess VTE complications prior to the start of cancer treatment. A 1-year follow-up was conducted to observe the incidence of events, such as VTE, cerebral infarction, bleeding, and death, during cancer treatment.

The present post hoc sub-analysis of the Cancer-VTE Registry aimed to determine the relationship between D-dimer level at the time of cancer diagnosis and the incidence of thrombotic events and mortality during the subsequent 1-year follow-up period (during cancer treatment) using a dataset from the Cancer-VTE Registry.

## Patients and methods

### Study design

This was a post hoc sub-analysis of the Cancer-VTE Registry. The study rationale and design details have been previously reported [15–17]. Briefly, the Cancer-VTE Registry was a nationwide, large-scale, multicenter prospective observational study conducted in Japan between March 2017 and February 2019, with a 1-year follow-up. The Non-Profit Organization Clinical Research Promotion Network Japan approved the protocol (approval number: not applicable). The study adhered to the Declaration of Helsinki and the Ethical Guidelines for Medical and Health Research Involving Human Subjects by the Japanese Ministry of Health, Labour and Welfare. All patients provided written informed consent. The Cancer-VTE Registry was registered at UMIN Clinical Trials Registry under the identifier number UMIN000024942.

### Patients

In the main study, cancer patients (colorectal, lung, stomach, pancreatic, breast, and gynecologic cancers) aged ≥ 20 years who were planned to initiate cancer therapy or first-line therapy for relapsed cancer were included [17]. Patients underwent VTE screening within 2 months prior to registration. However, if the D-dimer level measured after cancer diagnosis was ≤1.2 µg/mL, VTE screening was not required, and enrollment was allowed assuming that the patient did not have VTE at baseline [18]. Among the patients in the analysis set of the main study, those with measured D-dimer levels at baseline were included in this sub-analysis.

### Outcome measures

This study investigated the following: the distribution of D-dimer levels at baseline among cancer patients; patient characteristics prior to initiation of cancer treatment; the relationship between D-dimer levels at baseline and the incidence of events, including symptomatic VTE, incidental VTE requiring treatment, and composite VTE (symptomatic VTE and incidental VTE requiring treatment), cerebral infarction/transient ischemic attack (TIA)/systemic embolic events (SEE), bleeding (major or clinically relevant non-major bleeding), and all-cause death during the 1-year follow-up period of cancer treatment; and causes of death. Event risk ratios for D-dimer levels at baseline were also evaluated. A subgroup analysis of all-cause death according to baseline D-dimer level and cancer stage at baseline was conducted. Furthermore, a subgroup analysis of events according to baseline D-dimer level in patients with and without VTE at baseline was conducted.

### Statistical methods

As this was a post hoc analysis, the sample size was not pre-specified. For the main study, the sample size calculations and other details of the statistical analysis have been previously reported [15, 16]. Descriptive statistics were used to summarize patient characteristics, including mean ± standard deviation (SD) and median (minimum, maximum) for continuous data, and *n* (%) for categorical data. Event incidence (proportion of the patients experienced the event) was stratified by D-dimer level. Hazard ratios (HRs) of events during the 1-year follow-up period were calculated. HRs per 1.0-µg/mL increase in D-dimer level, with D-dimer level as a continuous variable, and HRs for two-category comparisons, with 1.2 µg/mL as the cutoff value, were determined. For events other than death during the follow-up period, HRs were calculated using the Fine and Gray model with death as a competing risk. For all-cause death, HRs were calculated using the Cox proportional hazards model. The variables for the multivariable analysis were set as the factors related to D-dimer level based on previous studies [19, 20] as follows: stage (I/IB/II, III, IV), age (<65, ≥65 years), renal function (creatinine clearance ≤ 50, >50 mL/min), Eastern Cooperative Oncology Group performance status (ECOG PS; 0, 1, 2), and presence of VTE at enrollment were used. Two-sided *P* values <0.05 were considered statistically significant. All statistical analyses were performed using SAS software version 9.4 (SAS Institute Inc., Cary, NC, USA).

## Results

### Patients

Of the 9630 patients in the main analysis, 610 patients whose D-dimer levels were not measured at baseline were excluded; therefore, 9020 patients were included in this analysis (Online Resource 1). Patient characteristics are summarized in Table 1. The mean ± SD age was 66.6 ± 11.9 years and 50.8% of patients were male. The most common cancer types were colorectal and lung cancer (25.3% each), followed by stomach cancer (18.2%), pancreatic cancer (10.9%), breast cancer (10.6%), and gynecologic cancer (9.7%). In total, 53.3% of patients had cancer stage III and IV, 74.1% had an ECOG PS of 0, and 5.8% had VTE at baseline.

**Table 1:** Patient characteristics

	All patients (*N* = 9020)
Male sex, *n* (%)	4582 (50.8)
Age, years, mean ± SD	66.6 ± 11.9
≥65 years, *n* (%)	5747 (63.7)
Cancer type, *n* (%)	
Colorectal	2279 (25.3)
Lung	2278 (25.3)
Stomach	1646 (18.2)
Pancreatic	986 (10.9)
Breast	955 (10.6)
Gynecologic	876 (9.7)
Cancer stage^a^, *n* (%)	
I/IB/II	4214 (46.7)
III	2648 (29.4)
IV	2158 (23.9)
ECOG PS^b^, *n* (%)	
0	6681 (74.1)
1	2018 (22.4)
2	321 (3.6)
VTE at baseline, *n* (%)	524 (5.8)
CrCL, mL/min, mean ± SD	78 ± 29
≤50 mL/min, *n* (%)	1201 (13.3)
Platelet count, × 10^9^/L, mean ± SD	267 ± 88
≥350 × 10^9^/L, *n* (%)	1280 (14.2)
Hb, g/dL, mean ± SD	12.7 ± 2.0
<10 g/dL, *n* (%)	841 (9.3)
WBC count, × 10^9^/L, mean ± SD	6.7 ± 2.6
>11 × 10^9^/L, *n* (%)	361 (4.0)

*CrCL* creatinine clearance; *ECOG PS* Eastern Cooperative Oncology Group performance status; *Hb* hemoglobin; *SD* standard deviation; *VTE* venous thromboembolism; *WBC* white blood cell

^a^Stage I is gynecologic cancer only, and stage IB is lung cancer only

^b^Pancreatic cancer is ECOG PS 0 and 1 only

### Distribution of baseline D-dimer levels

**Fig. 1:**
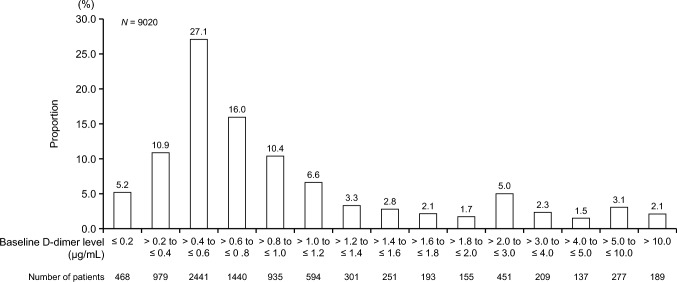
Distribution of baseline D-dimer levels in the whole population with measured D-dimer levels

The mean ± SD baseline D-dimer level was 1.57 ± 3.94 µg/mL (median [minimum, maximum]: 0.70 [0.0, 127.3] µg/mL). D-dimer levels ≤ 2.0 µg/mL were tabulated in 0.2-µg/mL increments (10 categories), D-dimer levels of >2.0 to ≤5.0 µg/mL were tabulated in 1.0-µg/mL increments (three categories), and D-dimer levels > 5.0 µg/mL were categorized as “>5.0 to ≤10.0 µg/mL” and “>10.0 µg/mL”, resulting in a total of 15 D-dimer level categories. The D-dimer level category with the highest proportion of patients was >0.4 to ≤0.6 µg/mL (27.1%) (Fig. 1).

### Incidence of thrombotic events during the follow-up period according to baseline D-dimer level

**Fig. 2:**
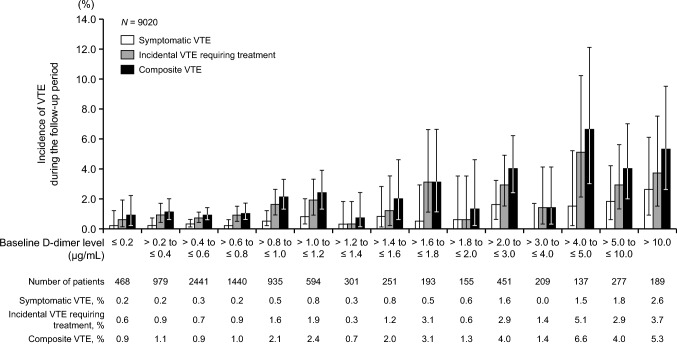
Incidences of symptomatic VTE, incidental VTE requiring treatment, and composite VTE during the follow-up period according to baseline D-dimer level (*N* = 9020). Error bars indicate 95% confidence intervals. Composite VTE consists of symptomatic VTE and incidental VTE requiring treatment. VTE, venous thromboembolism

**Fig. 3:**
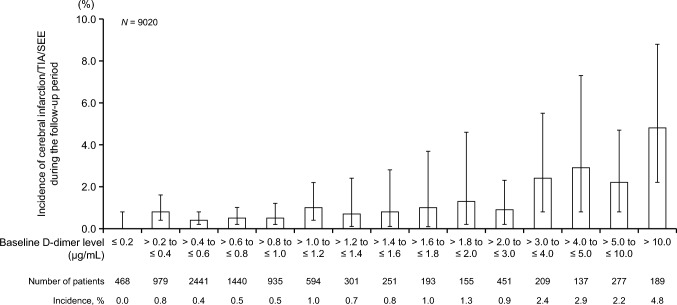
Incidence of cerebral infarction/TIA/SEE during the follow-up period according to baseline D-dimer level (*N* = 9020). Error bars indicate 95% confidence intervals. SEE, systemic embolic events; TIA, transient ischemic attack

**Fig. 4:**
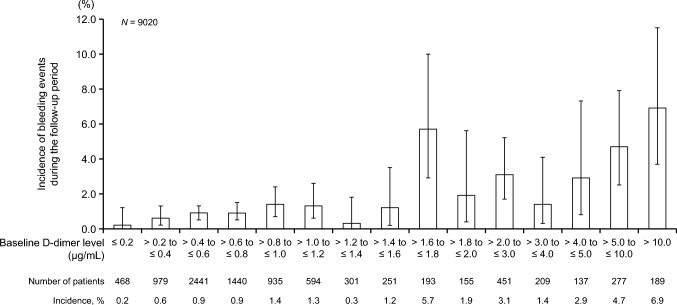
Incidence of bleeding during the follow-up period according to baseline D-dimer level (*N* = 9020). Bleeding includes major bleeding and clinically relevant non-major bleeding events. Error bars indicate 95% confidence intervals

The incidence of events (VTE, cerebral infarction/TIA/SEE, bleeding) during the follow-up period according to baseline D-dimer level is shown in Figs. 2, 3 and 4. The incidences of symptomatic VTE, incidental VTE requiring treatment, composite VTE, cerebral infarction/TIA/SEE, and bleeding events tended to increase with increasing baseline D-dimer level during the follow-up period. The incidence of thrombotic events according to baseline D-dimer level in patients with and without VTE at baseline is shown in Online Resources 2 and 3.

### Baseline D-dimer level and all-cause death

**Fig. 5:**
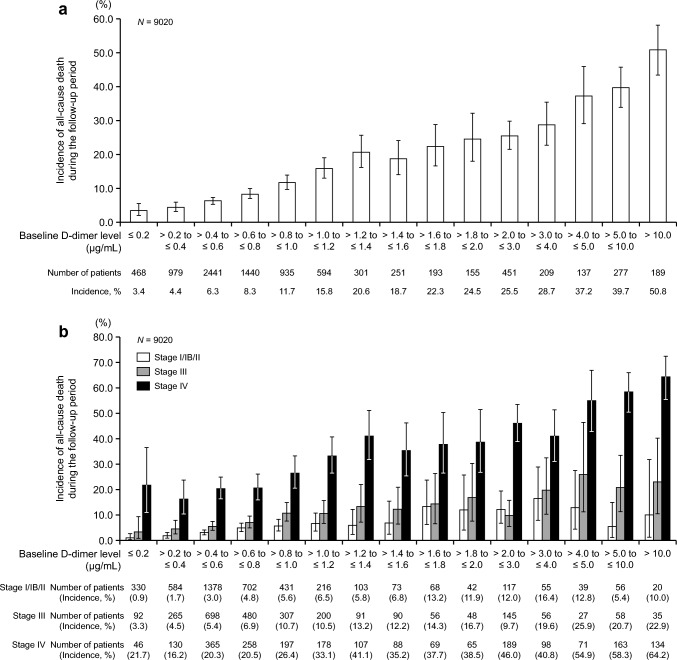
Incidence of all-cause death during the follow-up period according to baseline D-dimer level (**a**) and stratified by cancer stage (**b**). Stage I is for gynecologic cancer only, and IB is for lung cancer only. Error bars indicate 95% confidence intervals

The incidence of all-cause death according to baseline D-dimer level is shown in Fig. 5a, and that stratified by cancer stage is shown in Fig. 5b. The incidence of all-cause death tended to increase during the follow-up period, with increasing baseline D-dimer level, and this trend was generally maintained regardless of the cancer stage. A similar trend was observed for patients both with and without VTE at baseline (Online Resources 2, 3). Of the 1157 patients who died, cancer itself accounted for most deaths (Online Resource 4).

### Baseline D-dimer level and risk of events during the follow-up period

Table 2 shows the univariable and multivariable analyses of the event HRs per 1.0-µg/mL increase in baseline D-dimer level. The adjusted HRs were statistically significant for the following events: symptomatic VTE, 1.03 (95% confidence interval [CI], 1.00–1.05); incidental VTE requiring treatment, 1.02 (95% CI, 1.01–1.04); composite VTE, 1.02 (95% CI, 1.01–1.04); cerebral infarction/TIA/SEE, 1.04 (95% CI, 1.02–1.05); and all-cause death, 1.03 (95% CI, 1.02–1.03). The incidences and risks of events during the follow-up period according to a baseline D-dimer level cutoff of 1.2 µg/mL are shown in Online Resource 5. As with the results of event risk per 1.0-µg/mL increment in baseline D-dimer level, the results showed significant risks for all events except for bleeding.

**Table 2:** Incidence and risk of events during the follow-up period per 1.0-µg/mL increase in baseline D-dimer level

Events	Incidence *n* (%) [95% CI]	Univariable		Multivariable^c^	
		HR [95% CI]	*P* value	HR [95% CI]	*P* value
Symptomatic VTE	47 (0.5) [0.4–0.7]	1.04 [1.02–1.05]	<0.001	1.03 [1.00–1.05]	0.018
Incidental VTE requiring treatment	116 (1.3) [1.1–1.5]	1.03 [1.02–1.04]	<0.001	1.02 [1.01–1.04]	0.008
Composite VTE^a^	153 (1.7) [1.4–2.0]	1.03 [1.02–1.04]	<0.001	1.02 [1.01–1.04]	0.005
Cerebral infarction/TIA/SEE	72 (0.8) [0.6–1.0]	1.04 [1.03–1.05]	<0.001	1.04 [1.02–1.05]	<0.001
Bleeding^b^	127 (1.4) [1.2–1.7]	1.04 [1.02–1.05]	<0.001	1.01 [0.99–1.03]	0.359
All-cause death	1157 (12.8) [12.1–13.5]	1.05 [1.04–1.05]	<0.001	1.03 [1.02–1.03]	<0.001

*CI* confidence interval; *ECOG PS* Eastern Cooperative Oncology Group performance status; *HR* hazard ratio; *SEE* systemic embolic events; *TIA* transient ischemic attack; *VTE* venous thromboembolism

^a^A composite of symptomatic VTE events and incidental VTE events requiring treatment

^b^Included major bleeding and clinically relevant non-major bleeding events

^c^Adjusted by stage (I/IB/II, III, IV), age (<65, ≥65 years), renal function (creatinine clearance of ≤50, >50 mL/min), ECOG PS (0, 1, 2), VTE at baseline (yes, no)

## Discussion

The Cancer-VTE Registry is a large-scale prospective cohort study that has collected data on VTE prevalence in Japanese cancer patients under routine clinical practice. This sub-analysis of the Cancer-VTE Registry found that a higher baseline D-dimer level was a significant risk factor for thrombotic events, such as VTE and cerebral infarction, but also for death during the follow-up period.

In the present study, higher D-dimer levels at baseline were associated with more thrombotic events in the subsequent 1-year follow-up period. This was consistent with a previous study reporting that preoperative D-dimer levels were associated with a higher incidence of postoperative deep vein thrombosis in patients undergoing colorectal cancer surgery [21]. Indeed, some VTE prediction scores exist for cancer patients that incorporate D-dimer level [22, 23]. One of these, the Vienna Cancer and Thrombosis Study (CATS) score [23], is a VTE prediction model for ambulatory cancer patients that combines only two factors: tumor site risk category (low-intermediate, high, or very high) and continuous D-dimer concentration. Patients with a VTE risk threshold of ≥8% according to the CATS score have been reported to experience improved efficiency of drug thromboprophylaxis [24].

By contrast, higher D-dimer levels at baseline were not associated with more bleeding events. In patients undergoing anticoagulant therapy after orthopedic surgery, it has been reported that D-dimer levels are high in patients with major bleeding [25]. However, there are no reports examining the relationship between high D-dimer levels and subsequent bleeding events. Although D-dimer reflects both hyper-fibrinolysis and hyper-coagulable state, the results of this study suggest that high D-dimer levels at the time of cancer diagnosis mainly reflect the hyper-coagulable state.

In the present study, a relationship between increasing D-dimer level and increasing mortality was observed in all cancer stages. Furthermore, thromboembolism-related death was not common in this study; more than 90% of deaths were due to cancer itself. Therefore, we believe that the increased D-dimer levels may predict worsening cancer disease status as well as VTE status.

The association between worsening cancer disease and D-dimer level is not necessarily associated with VTE. D-dimer level is positively correlated with cancer stage and metastasis [26]. In pancreatic cancer patients, a correlation between D-dimer levels and the likelihood of resection has been reported [13]. In colorectal cancer patients, D-dimer level was found to be associated with poor prognosis in those without VTE [27]. Finally, in patients with gastrointestinal cancer, D-dimer level was found to be associated with early disease progression after chemotherapy and death [28].

Increased D-dimer levels in cancer patients without the presence of VTE could be due to, at least in part, the increased coagulation activity by tumor cells [3], which is believed to play an important role in the mechanism of cancer metastasis [29–31]. Remote metastasis is initiated when the primary tumor releases tumor cells into the circulatory system to become circulating tumor cells. At this time, the coagulation cascade is activated, and a thrombus of multi-platelets forms around the tumor cells, protecting them from shear stress and lysis by natural killer cells [32]. The formation of thrombin is activated by a tissue factor expressed by tumor cells, leading to coagulation and platelet activation, both of which enhance tumor metastasis [32].

VTE is a well-known complication of cancer. The diagnosis of VTE requires imaging tests, such as lower extremity echo and contrast-enhanced computed tomography, but D-dimer is often measured first in routine practice. Performing imaging tests on all patients with suspected VTE is undesirable owing to medical costs, exposure to radiation, and the risk of contrast nephropathy [33]. Furthermore, a D-dimer test is a simple and inexpensive test that can be performed by blood sampling. Therefore, a combination of clinical symptoms and D-dimer level is commonly used to screen patients for imaging tests, and several clinical probability scores are also utilized [22, 23, 34–36].

Regarding the clinical implications of measuring D-dimer at the time of cancer diagnosis, the results of this study showed that a D-dimer level increment of 1.0 µg/mL was associated with a significantly higher risk of VTE, cerebral infarction/TIA/SEE, and all-cause death in the follow-up period during cancer treatment. Assessment of patient prognosis at the time of cancer diagnosis is clinically important for decision-making about subsequent treatment. D-dimer levels may reflect some factors that increase the risk of death, which are not included in the cancer stage classification, and may be a marker for improving the prognosis of cancer patients in future. However, the D-dimer cutoff values for events were not determined in this study. It should be noted that this study evaluated the relationship between D-dimer level and thrombotic events and mortality during cancer treatment based on the overall picture from a prospective study of a large number of cancer patients.

### Limitations

The present analysis is limited for several reasons. First, although D-dimer levels are known to vary depending on the measuring reagent used [37, 38], information on the reagent used at each facility was not collected. However, as the types of D-dimer test kits used were limited to those available in Japan, it is assumed that there was little variability in the reagents used in the present study. Second, a high D-dimer level at cancer diagnosis was associated with death regardless of cancer stage. However, the cancer stage distribution in the Cancer-VTE Registry varied among cancer types [15]. Third, absolute mortality rates vary widely among cancer types. The effect of cancer type on mortality is difficult to adjust for and was not evaluated in the present study; therefore, the relationship between D-dimer levels and events may differ among cancer types. Fourth, patients with a higher D-dimer level at enrollment had a higher prevalence of VTE at enrollment and a higher proportion of anticoagulant use. It is possible that anticoagulation might have affected outcomes during the follow-up period; however, this was not an adjustment factor in the present study, so bias exists. Finally, D-dimer values were collected only at baseline and not during the follow-up period, so it was not possible to evaluate changes in D-dimer by treatment and D-dimer levels at the time of event onset.

## Conclusion

The current study revealed that regardless of cancer stage, higher baseline D-dimer levels were associated with higher mortality. Increases in D-dimer levels were also associated with a higher risk of VTE, and cerebral infarction/TIA/SEE during cancer treatment. The measurement of D-dimer levels at the time of cancer diagnosis is useful for assessing the risk of mortality.

### Supplementary Information

Below is the link to the electronic supplementary material.

Supplementary file1 (DOCX 69 KB)

## Data Availability

The anonymized data underlying the results presented in this manuscript may be made available to researchers upon submission of a reasonable request to the corresponding author. The decision to disclose the data will be made by the corresponding author and the funder, Daiichi Sankyo Co., Ltd. The data disclosure can be requested for 36 months from the article publication.
