# Supplementary material for: Association of D-dimer level with thrombotic events, bleeding, and mortality in Japanese patients with solid tumors: a Cancer-VTE Registry subanalysis
Source: Int J Clin Oncol. 2024 Mar 2;29(4):407–16. doi: 10.1007/s10147-024-02475-6 (PMC10963516; doi:10.1007/s10147-024-02475-6)
Supplement: Supplementary file 1 — Supplementary file1 (DOCX 69 KB) [file 10147_2024_2475_MOESM1_ESM.docx]

***International Journal of Clinical Oncology***

**Online Resource**

**Association of D-dimer level with thrombotic events, bleeding, and mortality in Japanese patients with solid tumors: a Cancer-VTE Registry subanalysis**

Mashio Nakamura, Masato Sakon, Mitsuru Sasako, Takuji Okusaka, Hirofumi Mukai, Keiichi Fujiwara, Hideo Kunitoh, Mari S. Oba, Hideo Wada, Jun Hosokawa, Atsushi Takita, Masataka Ikeda

**Corresponding author**

Mashio Nakamura, MD, PhD

Nakamura Medical Clinic, 7-1510, Hidamarinooka, Kuwana, Mie 511-0867, Japan

Tel: +81 594-33-1616

E-mail: [nakamura@hidamari-naika.jp](mailto:nakamura@hidamari-naika.jp)


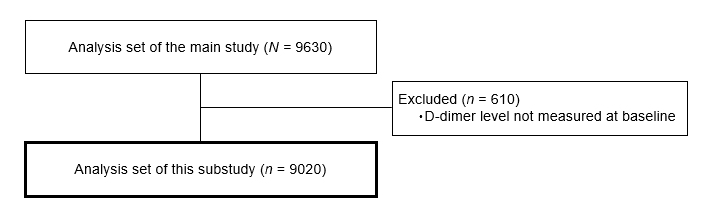


**Online resource 1.** Patient disposition

**Online resource 2.** Incidence of events during the follow-up period according to baseline D-dimer level in patients with VTE at baseline

(*n* = 524)

|  | **Baseline D-dimer level, µg/mL** | | | | | | | | | | | | | | |
| --- | --- | --- | --- | --- | --- | --- | --- | --- | --- | --- | --- | --- | --- | --- | --- |
|  | ≤ 0.2 (*n* = 2, 0.4%) | > 0.2  to ≤ 0.4 (*n* = 5, 1.0%) | > 0.4  to ≤ 0.6 (*n* = 9, 1.7%) | > 0.6  to ≤ 0.8 (*n* = 9, 1.7%) | > 0.8  to ≤ 1.0 (*n* = 10, 1.9%) | > 1.0 to  ≤ 1.2 (*n* = 12, 2.3%) | > 1.2 to  ≤ 1.4 (*n* = 34, 6.5%) | > 1.4  to ≤ 1.6 (*n* = 33, 6.3%) | > 1.6  to ≤ 1.8 (*n* = 34, 6.5%) | > 1.8 to ≤ 2.0 (*n* = 21, 4.0%) | > 2.0  to ≤ 3.0 (*n* = 98, 18.7%) | > 3.0  to ≤ 4.0 (*n* = 40, 7.6%) | > 4.0  to ≤ 5.0 (*n* = 34, 6.5%) | > 5.0  to ≤ 10.0  (*n* = 86, 16.4%) | > 10.0 (*n* = 97, 18.5%) |
| Symptomatic VTE | 0 (0.0) [0.0–84.2] | 0 (0.0) [0.0–52.2] | 0 (0.0) [0.0–33.6] | 0 (0.0) [0.0–33.6] | 0 (0.0) [0.0–30.8] | 0 (0.0) [0.0–26.5] | 0 (0.0) [0.0–10.3] | 0 (0.0) [0.0–10.6] | 0 (0.0) [0.0–10.3] | 1 (4.8) [0.1–23.8] | 3 (3.1) [0.6–8.7] | 0 (0.0) [0.0–8.8] | 1 (2.9) [0.1–15.3] | 2 (2.3) [0.3–8.1] | 4 (4.1) [1.1–10.2] |
| Incidental VTE requiring treatment | 0 (0.0) [0.0–84.2] | 0 (0.0) [0.0–52.2] | 0 (0.0) [0.0–33.6] | 0 (0.0) [0.0–33.6] | 0 (0.0) [0.0–30.8] | 0 (0.0) [0.0–26.5] | 0 (0.0) [0.0–10.3] | 0 (0.0) [0.0–10.6] | 3 (8.8) [1.9–23.7] | 1 (4.8) [0.1–23.8] | 5 (5.1) [1.7–11.5] | 0 (0.0) [0.0–8.8] | 2 (5.9) [0.7–19.7] | 1 (1.2) [0.0–6.3] | 6 (6.2) [2.3–13.0] |
| Composite VTE^a^ | 0 (0.0) [0.0–84.2] | 0 (0.0) [0.0–52.2] | 0 (0.0) [0.0–33.6] | 0 (0.0) [0.0–33.6] | 0 (0.0) [0.0–30.8] | 0 (0.0) [0.0–26.5] | 0 (0.0) [0.0–10.3] | 0 (0.0) [0.0–10.6] | 3 (8.8) [1.9–23.7] | 2 (9.5) [1.2–30.4] | 7 (7.1) [2.9–14.2] | 0 (0.0) [0.0–8.8] | 3 (8.8) [1.9–23.7] | 3 (3.5) [0.7–9.9] | 8 (8.2) [3.6–15.6] |
| Cerebral infarction/TIA/SEE | 0 (0.0) [0.0–84.2] | 0 (0.0) [0.0–52.2] | 0 (0.0) [0.0–33.6] | 0 (0.0) [0.0–33.6] | 0 (0.0) [0.0–30.8] | 0 (0.0) [0.0–26.5] | 0 (0.0) [0.0–10.3] | 0 (0.0) [0.0–10.6] | 0 (0.0) [0.0–10.3] | 0 (0.0) [0.0–16.1] | 0 (0.0) [0.0–3.7] | 0 (0.0) [0.0–8.8] | 1 (2.9) [0.1–15.3] | 2 (2.3) [0.3–8.1] | 8 (8.2) [3.6–15.6] |
| Bleeding^b^ | 0 (0.0) [0.0–84.2] | 1 (20.0) [0.5–71.6] | 0 (0.0) [0.0–33.6] | 0 (0.0) [0.0–33.6] | 0 (0.0) [0.0–30.8] | 1 (8.3) [0.2–38.5] | 1 (2.9) [0.1–15.3] | 2 (6.1) [0.7–20.2] | 4 (11.8) [3.3–27.5] | 0 (0.0) [0.0–16.1] | 3 (3.1) [0.6–8.7] | 1 (2.5) [0.1–13.2] | 1 (2.9) [0.1–15.3] | 9 (10.5) [4.9–18.9] | 8 (8.2) [3.6–15.6] |
| All-cause death | 0 (0.0) [0.0–84.2] | 2 (40.0) [5.3–85.3] | 2 (22.2) [2.8–60.0] | 1 (11.1) [0.3–48.2] | 1 (10.0) [0.3–44.5] | 1 (8.3) [0.2–38.5] | 5 (14.7) [5.0–31.1] | 9 (27.3) [13.3–45.5] | 10 (29.4) [15.1–47.5] | 2 (9.5) [1.2–30.4] | 22 (22.4) [14.6–32.0] | 10 (25.0) [12.7–41.2] | 11 (32.4) [17.4–50.5] | 42 (48.8) [37.9–59.9] | 50 (51.5) [41.2–61.8] |

^a^ A composite of symptomatic VTE events and incidental VTE events requiring treatment.

^b^ Included major bleeding and clinically relevant non-major bleeding events.

Data are *n* (%) [95%CI].

*CI* confidence interval; *SEE* systemic embolic event; *TIA* transient ischemic attack; *VTE* venous thromboembolism.

**Online resource 3.** Incidence of events during the follow-up period according to baseline D-dimer level in patients without VTE at baseline (*n* = 8496)

|  | **Baseline D-dimer level, µg/mL** | | | | | | | | | | | | | | |
| --- | --- | --- | --- | --- | --- | --- | --- | --- | --- | --- | --- | --- | --- | --- | --- |
|  | ≤ 0.2  (*n* = 466, 5.5%) | > 0.2 to  ≤ 0.4 (*n* = 974, 11.5%) | > 0.4 to  ≤ 0.6 (*n* = 2432, 28.6%) | > 0.6 to  ≤ 0.8 (*n* = 1431, 16.8%) | > 0.8 to  ≤ 1.0 (*n* = 925, 10.9%) | > 1.0 to  ≤ 1.2 (*n* = 582, 6.9%) | > 1.2 to  ≤ 1.4 (*n* = 267, 3.1%) | > 1.4 to  ≤ 1.6 (*n* = 218, 2.6%) | > 1.6 to  ≤ 1.8 (*n* = 159, 1.9%) | > 1.8 to  ≤ 2.0 (*n* = 134, 1.6%) | > 2.0 to  ≤ 3.0 (*n* = 353, 4.2%) | > 3.0 to  ≤ 4.0 (*n* = 169, 2.0%) | > 4.0 to ≤ 5.0 (*n* = 103, 1.2%) | > 5.0  to ≤ 10.0 (*n* = 191, 2.2%) | > 10.0   (*n* = 92, 1.1%) |
| Symptomatic VTE | 1 (0.2) [0.0–1.2] | 2 (0.2) [0.0–0.7] | 7 (0.3) [0.1–0.6] | 3 (0.2) [0.0–0.6] | 5 (0.5) [0.2–1.3] | 5 (0.9) [0.3–2.0] | 1 (0.4) [0.0–2.1] | 2 (0.9) [0.1–3.3] | 1 (0.6) [0.0–3.5] | 0 (0.0) [0.0–2.7] | 4 (1.1) [0.3–2.9] | 0 (0.0) [0.0–2.2] | 1 (1.0) [0.0–5.3] | 3 (1.6) [0.3–4.5] | 1 (1.1) [0.0–5.9] |
| Incidental VTE requiring treatment | 3 (0.6) [0.1–1.9] | 9 (0.9) [0.4–1.7] | 16 (0.7) [0.4–1.1] | 13 (0.9) [0.5–1.5] | 15 (1.6) [0.9–2.7] | 11 (1.9) [0.9–3.4] | 1 (0.4) [0.0–2.1] | 3 (1.4) [0.3–4.0] | 3 (1.9) [0.4–5.4] | 0 (0.0) [0.0–2.7] | 8 (2.3) [1.0–4.4] | 3 (1.8) [0.4–5.1] | 5 (4.9) [1.6–11.0] | 7 (3.7) [1.5–7.4] | 1 (1.1) [0.0–5.9] |
| Composite VTE^a^ | 4 (0.9) [0.2–2.2] | 11 (1.1) [0.6–2.0] | 23 (0.9) [0.6–1.4] | 15 (1.0) [0.6–1.7] | 20 (2.2) [1.3–3.3] | 14 (2.4) [1.3–4.0] | 2 (0.7) [0.1–2.7] | 5 (2.3) [0.7–5.3] | 3 (1.9) [0.4–5.4] | 0 (0.0) [0.0–2.7] | 11 (3.1) [1.6–5.5] | 3 (1.8) [0.4–5.1] | 6 (5.8) [2.2–12.2] | 8 (4.2) [1.8–8.1] | 2 (2.2) [0.3–7.6] |
| Cerebral infarction/TIA/SEE | 0 (0.0) [0.0–0.8] | 8 (0.8) [0.4–1.6] | 10 (0.4) [0.2–0.8] | 7 (0.5) [0.2–1.0] | 5 (0.5) [0.2–1.3] | 6 (1.0) [0.4–2.2] | 2 (0.7) [0.1–2.7] | 2 (0.9) [0.1–3.3] | 2 (1.3) [0.2–4.5] | 2 (1.5) [0.2–5.3] | 4 (1.1) [0.3–2.9] | 5 (3.0) [1.0–6.8] | 3 (2.9) [0.6–8.3] | 4 (2.1) [0.6–5.3] | 1 (1.1) [0.0–5.9] |
| Bleeding^b^ | 1 (0.2) [0.0–1.2] | 5 (0.5) [0.2–1.2] | 21 (0.9) [0.5–1.3] | 13 (0.9) [0.5–1.5] | 13 (1.4) [0.8–2.4] | 7 (1.2) [0.5–2.5] | 0 (0.0) [0.0–1.4] | 1 (0.5) [0.0–2.5] | 7 (4.4) [1.8–8.9] | 3 (2.2) [0.5–6.4] | 11 (3.1) [1.6–5.5] | 2 (1.2) [0.1–4.2] | 3 (2.9) [0.6–8.3] | 4 (2.1) [0.6–5.3] | 5 (5.4) [1.8–12.2] |
| All-cause death | 16 (3.4) [2.0–5.5] | 41 (4.2) [3.0–5.7] | 151 (6.2) [5.3–7.2] | 119 (8.3) [6.9–9.9] | 108 (11.7) [9.7–13.9] | 93 (16.0) [13.1–19.2] | 57 (21.3) [16.6–26.8] | 38 (17.4) [12.6–23.1] | 33 (20.8) [14.7–27.9] | 36 (26.9) [19.6–35.2] | 93 (26.3) [21.8–31.3] | 50 (29.6) [22.8–37.1] | 40 (38.8) [29.4–48.9] | 68 (35.6) [28.8–42.8] | 46 (50.0) [39.4–60.6] |

^a^ A composite of symptomatic VTE events and incidental VTE events requiring treatment.

^b^ Included major bleeding and clinically relevant non-major bleeding events.

Data are *n* (%) [95%CI].

*CI* confidence interval; *SEE* systemic embolic event; *TIA* transient ischemic attack; *VTE* venous thromboembolism.

**Online resource 4.** Causes of death

|  | All patients  (*N* = 9020) |
| --- | --- |
|  |  |
| All-cause death | 1157 |
| Causes of death |  |
| Cancer | 1046 |
| VTE-related | 3 |
| Cerebral infarction/SEE | 10 |
| Bleeding | 5 |
| Others | 93 |

Data are *n*.

*SEE* systemic embolic event; *VTE* venous thromboembolism.

**Online resource 5.** Incidence and risk of events during the follow-up period according to a baseline D-dimer level cutoff of 1.2 µg/mL (*N* = 9020)

| Events | Baseline D-dimer level | | Univariable | | Multivariable^c^ | |
| --- | --- | --- | --- | --- | --- | --- |
|  | ≤ 1.2 µg/mL  (*n* = 6857) | > 1.2 µg/mL  (*n* = 2163) |  |  |  |  |
|  | Incidence,  n (%) [95% CI] | Incidence,  *n* (%) [95% CI] | HR [95% CI] | *P-*value | HR [95% CI] | *P-*value |
| Symptomatic VTE | 23 (0.3) [0.2–0.5] | 24 (1.1) [0.7–1.6] | 3.38 [1.91–5.98] | < 0.001 | 2.55 [1.25–5.20] | 0.010 |
| Incidental VTE requiring treatment | 67 (1.0) [0.8–1.2] | 49 (2.3) [1.7–3.0] | 2.37 [1.64–3.43] | < 0.001 | 1.93 [1.22–3.05] | 0.005 |
| Composite VTE^a^ | 87 (1.3) [1.0–1.6] | 66 (3.1) [2.4–3.9] | 2.47 [1.79–3.40] | < 0.001 | 1.94 [1.30–2.90] | 0.001 |
| Cerebral infarction/TIA/SEE | 36 (0.5) [0.4–0.7] | 36 (1.7) [1.2–2.3] | 3.24 [2.04–5.15] | < 0.001 | 2.18 [1.20–3.96] | 0.011 |
| Bleeding^b^ | 62 (0.9) [0.7–1.2] | 65 (3.0) [2.3–3.8] | 3.42 [2.42–4.84] | < 0.001 | 1.60 [0.98–2.60] | 0.058 |
| All-cause death | 535 (7.8) [7.2–8.5] | 622 (28.8) [26.9–30.7] | 4.42 [3.94–4.96] | < 0.001 | 2.24 [1.96–2.57] | < 0.001 |

^a^ A composite of symptomatic VTE and incidental VTE requiring treatment.

^b^ Included major bleeding and clinically relevant non-major bleeding.

^c^ Adjusted by Stage (I/IB/II, III, IV), age (< 65, ≥ 65 years), renal function (creatinine clearance of ≤ 50, > 50 mL/min), ECOG PS (0, 1, 2), VTE at baseline (yes, no).

*CI* confidence interval; *HR* hazard ratio; *SEE* systemic embolic event; *TIA* transient ischemic attack; *VTE* venous thromboembolism.
